# Case Report: Upper limb dysfunction may be caused by chest wall mass excision: An enlightenment from a special case

**DOI:** 10.3389/fonc.2022.947055

**Published:** 2022-08-03

**Authors:** Ping-Shang Wu, Ling Yuan, Dan Xiong, Yan-Hong Gao, Luan Xiang

**Affiliations:** ^1^ Department of Thoracic Cardiovascular Surgery, The Third Hospital of Wuhan, Wuhan, China; ^2^ Department of Pathology, General Hospital of Central Theater Command of the People’s Liberation Army, Wuhan, China; ^3^ Department of Cadre Ward First, General Hospital of Central Theater Command of the People’s Liberation Army, Wuhan, China; ^4^ Department of Ultrasound, General Hospital of Central Theater Command of The People’s Liberation Army, Wuhan, China; ^5^ Department of Thoracic Cardiovascular Surgery, General Hospital of Central Theater Command of the People’s Liberation Army, Wuhan, China

**Keywords:** schwannoma, surgical excision, lymph node, axilla, surgery trap, case report

## Abstract

Of all the thoracic surgical procedures, chest wall surgery is probably the lowest-risk type. In fact, it is not so. Clinical work also often has the trap of chest wall surgery. An operation to remove a mass in the axilla may result in upper limb disability on the affected side. Here, we report the case of a 47-year-old female patient with a left chest wall adjacent axillary mass, which was considered an abnormal structural lymph node on color ultrasound examination and chest CT. Otherwise, she felt no discomfort. The left upper limb moved freely without being affected by the mass. A routine resection of the tumor was performed after the preoperative examination was completed. After the operation, the incision recovered well. However, the day after the surgery, she developed numbness and pain in her left little finger and ring finger, pain that often kept her from sleeping. The mass was confirmed to be a schwannoma with cystic degeneration by pathology slicing after the operation. By this time, doctors were alerted to the fact that the removal of the chest wall mass had nearly disabled the left upper limb of the patient, which was a great warning to the thoracic surgeon. In this case report, we hope that all surgeons will be cautious and careful and will not trust the imaging diagnosis too much. It is also hoped that the patient understands that some procedures may lead to unexpected complications.

## Introduction

Schwannomas are tumors arising from the embryonic neural crest cells of the nerve sheaths of peripheral and cranial nerves, and they are a rare type of soft tissue mass, accounting for approximately 8% of all tumors (1). It mainly affects the head, neck, and flexor aspects of the limbs, where many nerves are distributed (1). A schwannoma presenting as a brachial plexus mass accounts for only approximately 5% of all schwannomas, and chest wall adjacent axillary schwannomas are extremely uncommon (2, 3). Surgeons, especially cardiothoracic surgeons, tend not to be afraid of complex surgery (4–7). What frustrates cardiothoracic surgeons are the unexpected complications of seemingly simple operations (8, 9). Some of the usual operations that do not enter the chest wall are often performed by the attending physician or resident alone. Permanent neurological deficits after the resection of upper limb masses have been reported (10). Serious complications can also occur during chest wall operations. The authors have heard of a few patients losing upper limb function on the affected side after a surgeon-performed excision of a chest wall mass. Here, the authors report their lessons from a case of chest cavity mass resection.

## Case report

**Figure 1:**
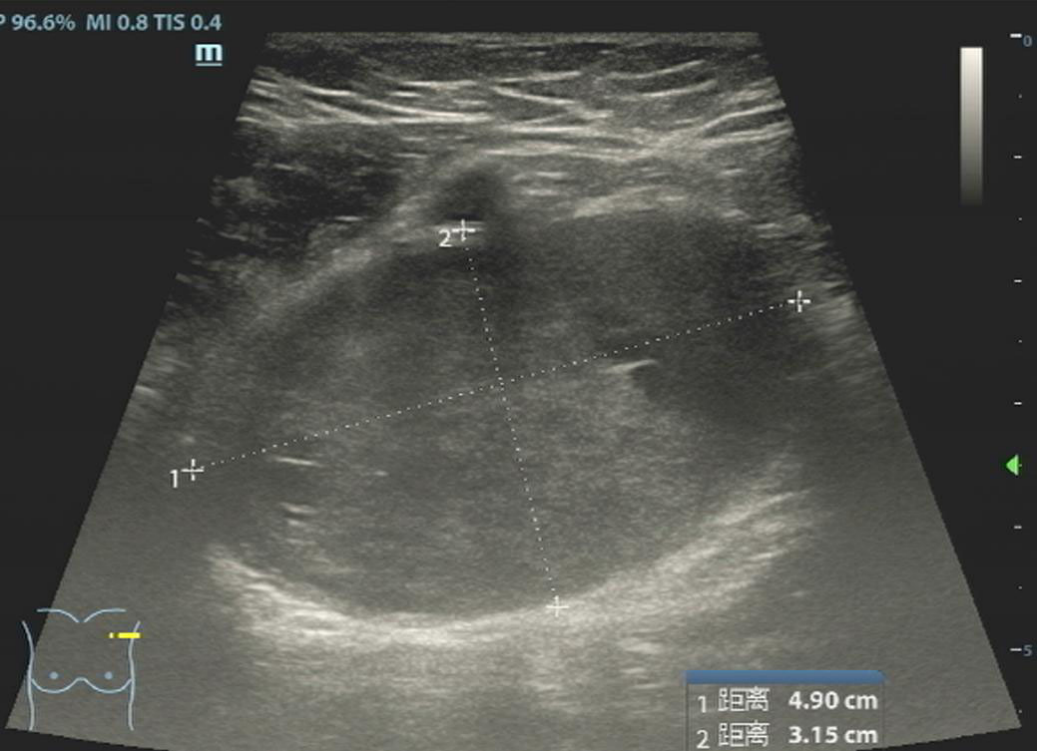
Ultrasound of the left axillary area showed a solid mass with two small cystic degenerations, which were highly suspected to be a few liquefied abnormal structures in the lymph nodes.

**Figure 2:**
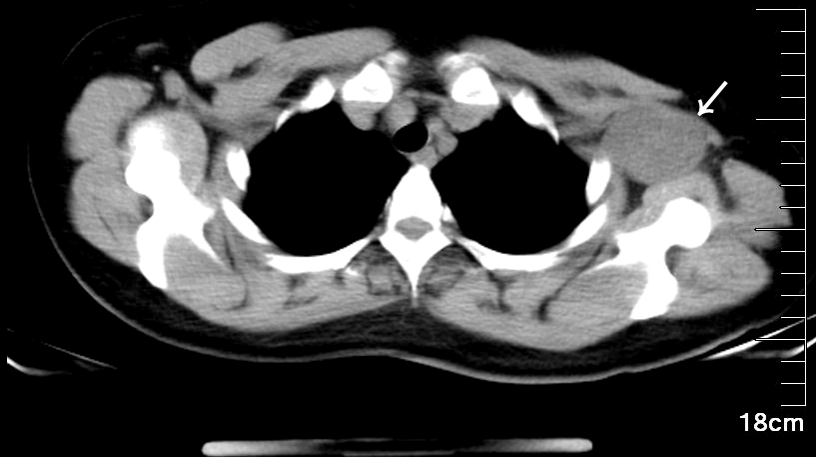
A mediastinal window with 16-slice spiral CT showed that the left axilla had a slightly circular and low-density shadow, with a cross-sectional area of approximately 33 mm × 47 mm and a CT value of approximately 33 HU. The mass was closely related to the chest wall, with a slender pedicle faintly visible.

**Figure 3:**
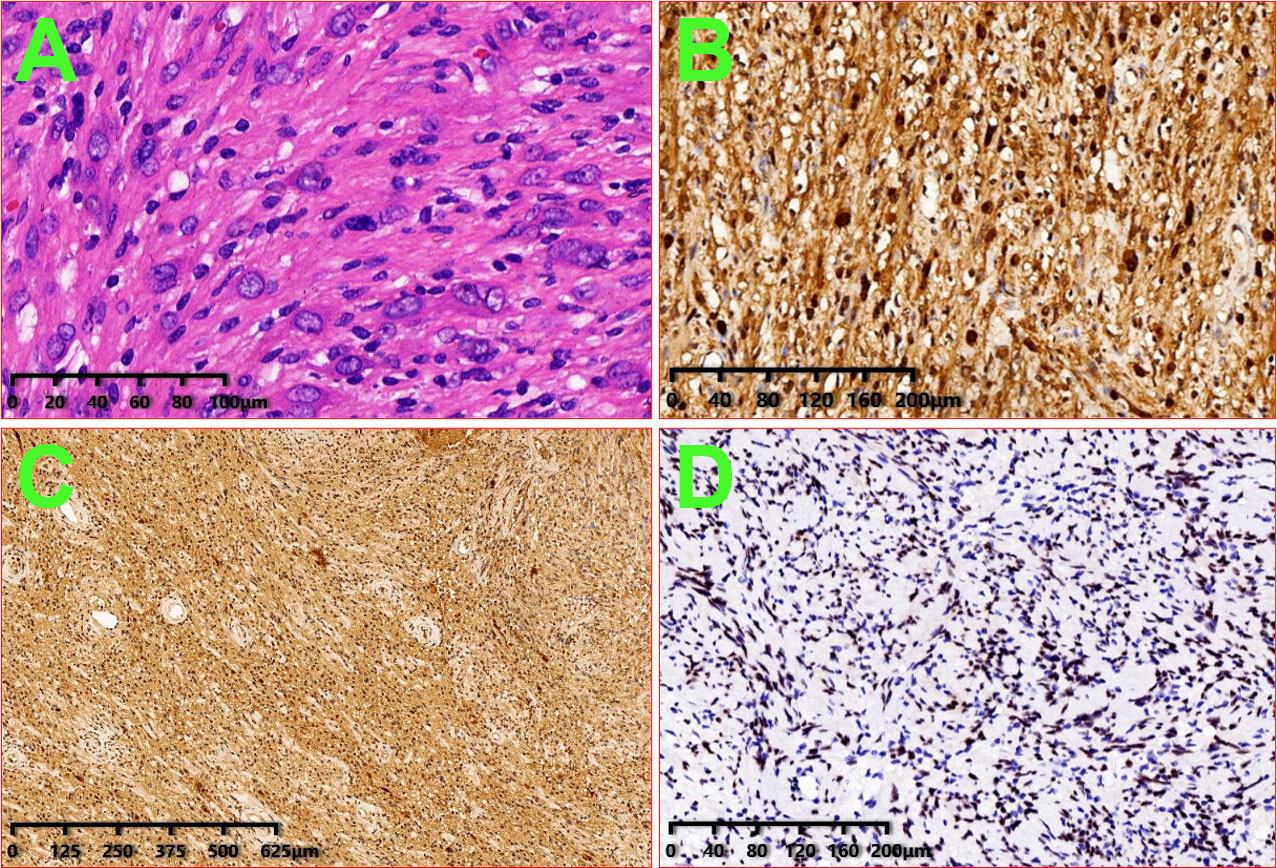
Image of histologic diagnosis from the left axillary mass. **(A)**: HE × 200; **(B)**: S100 × 100; **(C)**: PGP9.5 × 40; and **(D)**: SOX10 × 100.

A 47-year-old female patient was admitted to the hospital because of the discovery of a left chest wall adjacent axillary mass six months earlier. She had two children more than 20 years ago, and all of them were breastfed. She also had a left chest wall adjacent axillary mass three years ago, which had disappeared after two weeks (according to the own recollection of the patient). She had no prior history of tumors or breast nodules. Recently, she had no fever or other discomfort, and no local skin redness or swelling. Physical examination showed a palpable left chest wall adjacent axillary mass with well-defined boundaries, poor mobility, a hard, smooth surface, no tenderness, and no abnormal movement of the left upper limb. Color ultrasound examination indicated that a 4.9 × 3.2 cm solid hypoechoic nodule was seen in the left chest wall adjacent to the axilla, with clear boundaries, uneven internal echogenicity, and multiple echogenicity areas (**Figure 1**). CDFI: The above solid hypoechoic nodules show a visible blood flow signal. The sonographer identified this as an abnormal structure of the lymph nodes. Fine-needle histopathological examination showed spindle cells with focal, minimal atypia and a few fragments of fibrous tissue, suggestive of a cellular spindle lesion. Color ultrasound examination showed no obvious nodules in either breast or no obvious enlarged lymph nodes in the supraclavicular fossae and neck. Chest CT indicated that the left chest wall adjacent axillary had a slightly circular and low-density shadow, with a cross-sectional area of approximately 33 mm × 47 mm and a CT value of approximately 33 HU (**Figure 2**). No nodules were observed in either lung. No abnormalities were found in the ribs. A magnetic resonance imaging examination showed no abnormal lesions in the head or neck. Blood tests showed normal white blood cells, normal procalcitonin, and negative T-SOP. Her PPD test was also negative. After two weeks of follow-up, there was no significant change in the axillary mass. The patient desired surgical excision of the left chest wall and adjacent axillary mass after adequate communication by her doctor. During the operation, the left chest wall adjacent axillary masses were found under the skin with clear boundaries, poor mobility, complete envelop, and hard quality. It was carefully exfoliated along with the capsule. Then, the mass pedicle was found on the dorsal side of the mass, with a slight nerve distribution. The pedicle of the mass did not dare to be cut off with the careful removal of the mass, leaving a small capsule. After the operation, the patient developed numbness and pain in the left pinky and ring fingers, a pain that often kept her from sleeping. Fortunately, her left fist movement and grip strength were normal. These symptoms were slightly relieved by oral neurotrophic drugs and acupuncture. By this point in the description of the case, you may have assumed the pathological results of the operation. Yes, axillary schwannoma was confirmed by the histopathological section. It is worth mentioning that this is the rarest of the schwannomas, which are schwannomas with cystic lesions (**Figure 3**). After a year of follow-up, her symptoms of numbness and pain in the left pinky and ring fingers disappeared.

## Discussion

An axillary mass may appear in males and females, old and young individuals, and every racial individual (11). Common nonneoplastic diseases with chest wall adjacent axillary masses include lymphadenitis, sebaceous gland inflammatory masses, accessory breasts, lymph node tuberculosis, etc. Neoplastic axillary masses are commonly seen in lipoma, fibroma, accessory breast fibroadenoma, lymphangioma, hemangioma, skin apocrine carcinoma, sarcoma, lymph node metastatic carcinoma, etc. Chest wall adjacent axillary schwannoma is rarely reported in the literature (12, 13).

A schwannoma is a tumor originating from Schwann cells of the nerve sheath (14). It can affect all body parts, but it is most localized in the head and neck, and in the flexor aspect of the limbs, it can also localize, where many nerves are distributed (15, 16). Chest walls with adjacent axillary schwannomas are extremely uncommon. Chest wall adjacent axillary schwannomas may originate from the medial thoracic nerve, the medial thoracic nerve, the long thoracic nerve, and the costal interstitial nerve of the thoracic dorsal nerve. The apex of axillary schwannomas may also originate from abnormal movement of the ulnar nerve, medial cutaneous nerve, and other brachial plexus nerves.

In the absence of an obvious inflammatory lesion, a persistent axillary mass among adult females is frequently the first sign of breast cancer. Therefore, breast ultrasonography should be the primary adjunctive test for axillary masses in Asian populations or in patients with small breast glands. However, no matter what kind of imaging examination method is used, a satisfactory diagnostic coincidence rate cannot be achieved (17–20). Thus, because of their rare occurrence and involvement of unusual sites, they pose a significant challenge for diagnosis. The main reason he was misdiagnosed by ultrasound was that the schwannoma had undergone an extremely rare change—with cystic change. Schwannoma may have degenerative changes, such as hemorrhage, calcification, and fibrosis, but cystic changes are extremely rare (21, 22).

For such a rare case, which was misdiagnosed by ultrasound and could not be confirmed by fine-needle histopathological examination, the only reminder of the surgeon was some small amount of nerve distribution on the dorsal side of the mass during the operation. The outcomes of surgical treatment for chest wall masses presented in our case report are consistent with most literature about schwannomas of the upper extremities (14, 23, 24). During surgery, no visible injury to the nerve occurred, but nonetheless, its function was compromised. However, the literature confirms the possibility of such a scenario, especially in the case of motor nerve tumors. If these nerves are deeply hidden, they can also be cut by the surgeon. After surgery, the patient may no longer have upper limb function. Even if schwannoma is diagnosed before surgery, elective surgery is the best option for treating schwannomas and includes dissection and excision of the tumor from the nerve. In most cases, the tumor has a well-developed capsule, and the nerves do not penetrate it but ensnare it instead, thus allowing enucleation without damaging the bundles (14). Nerve bundles can sometimes penetrate tumors and need to be removed as well. Neurological deficits do not always occur after the excision of a single nerve bundle penetrating a tumor. This may be because the nerve bundles are not involved in the conduction.

There are limitations to this case report, particularly when examining it from a surgical standpoint. First, the preparation before surgery was inadequate, and the patient was not given magnetic resonance imaging before surgery. Second, the surgeon thinks it is just a lump, a superficial lump that grows above the chest wall, which is somewhat disrespectful. That means surgeons must adhere to scientific clinical practices and not disregard the condition of the patient just because they see a lump.

## Conclusion

Finally, we want to employ this case report to increase the number of medical workers studying chest wall masses who do not believe in medical imaging examination, careful exploration in the surgical process, more doubt, and more care. Let the public understand that there are many surgical traps in surgery to gain a better understanding of medical work.

## Data availability statement

The original contributions presented in the study are included in the article/supplementary material. Further inquiries can be directed to the corresponding author/s.

## Ethics statement

Ethical review and approval was not required for the study on human participants in accordance with the local legislation and institutional requirements. The patients/participants provided their written informed consent to participate in this study. Written informed consent was obtained from the individual(s) for the publication of any potentially identifiable images or data included in this article.

## Author contributions

P-SW, LY, and DX contributed equally to this report; therefore, they are considered co-first authors. They made a substantial contribution to the acquisition, data interpretation and draft manuscript preparation. Y-HG and LX critically studied the conception and designed and prepared and revised the manuscript for important intellectual content. All authors listed have made a substantial, direct, and intellectual contribution to the work and approved it for publication.

## Acknowledgments

The authors thank the patient who agreed to be included in this report for her cooperation and support for academic communication.

## Conflict of interest

The authors declare that the research was conducted in the absence of any commercial or financial relationships that could be construed as a potential conflict of interest.

## Publisher’s note

All claims expressed in this article are solely those of the authors and do not necessarily represent those of their affiliated organizations, or those of the publisher, the editors and the reviewers. Any product that may be evaluated in this article, or claim that may be made by its manufacturer, is not guaranteed or endorsed by the publisher.
